# Pre-anthesis light signaling of sheathed developing barley inflorescences defines floral fate

**DOI:** 10.1093/jxb/erag216

**Published:** 2026-05-13

**Authors:** Sai Thejas Babanna, Yongyu Huang, Andreas P M Weber, Thorsten Schnurbusch

**Affiliations:** Leibniz Institute of Plant Genetics and Crop Plant Research (IPK), Seeland 06466, Germany; Leibniz Institute of Plant Genetics and Crop Plant Research (IPK), Seeland 06466, Germany; Institute for Plant Biochemistry, Heinrich Heine- University, Duesseldorf 40225, Germany; Cluster of Excellence on Plant Sciences (CEPLAS) ‘SMART Plants for Tomorrow’s Needs’, Germany; Leibniz Institute of Plant Genetics and Crop Plant Research (IPK), Seeland 06466, Germany; Cluster of Excellence on Plant Sciences (CEPLAS) ‘SMART Plants for Tomorrow’s Needs’, Germany; Faculty of Natural Sciences III, Institute of Agricultural and Nutritional Sciences, Martin Luther University Halle-Wittenberg, Halle 06120, Germany; The University of Adelaide, Australia

**Keywords:** Dark treatment, maximum yield potential, pre-anthesis inflorescence greening, pre-anthesis tip degeneration, spikelet fertility, spikelet survival

## Abstract

Pre-anthesis inflorescence greening (PAIG) is a distinctive developmental feature in members of the *Triticeae* such as barley, wheat, and rye. In barley (*Hordeum vulgare* L.), floral survival and fertility are major determinants of grain yield, yet the physiological processes supporting early inflorescence development remain poorly understood. Here, we investigated PAIG, a light-dependent chlorophyll accumulation occurring while immature inflorescences are still enclosed by leaf sheaths, and its role in overall inflorescence development. Using chlorophyll autofluorescence imaging and chlorophyll quantification, we found that PAIG in barley initiates at a surprisingly early developmental stage, when developing spikes are still enclosed by leaf sheaths. PAIG first appears in the developing central rachis and then progressively spreads to the spikelet primordia and other floral organs. Using a non-destructive dark treatment that prevents light exposure to developing inflorescences, we showed that inhibiting PAIG does not impact floral initiation. Yet, the dark treatment significantly decreases floral survival and pollen viability, especially at the tip of the inflorescence. This highlights the essential role of light-mediated PAIG in determining floral fate. Finally, analysis of natural variation for PAIG revealed a strong positive correlation between the extent of PAIG and floral survival. Our findings establish PAIG as an underappreciated hidden trait that supports floral viability and reproductive success, with implications for enhancing grain yield potential in cereals.

## Teaser

Pre-anthesis inflorescence greening begins early in barley spikes within leaf sheaths. This light-dependent chlorophyll accumulation supports floral survival and pollen viability, revealing a previously overlooked trait influencing reproductive success and grain yield.

## Introduction

Light is a central regulator of plastid differentiation, photosynthetic gene expression, and chlorophyll biosynthesis (Pogson *et al*., 2015; Cackett *et al*., 2022). It also strongly affects reproductive development, with light intensity, photoperiod, and light quality influencing overall floral fertility (González *et al*., 2003, 2005; Parrado *et al*., 2025a, b). Several shading experiments clearly demonstrate that reduced irradiance during reproductive development can significantly diminish grain yield (Slafer *et al*., 1994; Dong *et al*., 2019; Golan *et al*., 2023), largely due to insufficient assimilate supply and impaired source–sink balance (Toyota *et al*., 2001; Yang *et al*., 2020). By regulating chloroplast development and reproductive organ formation, light directly influences the photosynthetic activity of the inflorescence, which supports grain development.

In cereals, fully developed inflorescence organs including awns, lemma, palea, and glumes containing functional chloroplasts have been shown to significantly contribute to the final grain yield, especially in the later stages of development when the leaves start to senesce (McKenzie, 1972; Imaizumi *et al*., 1990; Gebbing and Schnyder, 2001; Sanchez-Bragado *et al*., 2016). These chlorophyllous reproductive structures supply assimilates to developing grains and other sink tissues within the spike (Li *et al*., 2006; AuBuchon-Elder *et al*., 2020). In *Triticeae* species, such as barley (*Hordeum vulgare* L.), wheat (*Triticum aestivum* L.), and rye (*Secale cereale* L.), chlorophyll accumulation in the inflorescence begins at an early developmental stage, prior to anthesis, while the inflorescence is still enclosed by multiple layers of leaf sheaths (Huang *et al*., 2023; Shanmugaraj *et al*., 2023). Pre-anthesis inflorescence greening (PAIG) is a characteristic feature of *Triticeae* species and occurs already well before emergence (Huang *et al*., 2023).

Inflorescence development is a major determinant of reproductive success in cereals. Barley has a spike-type inflorescence in which spikelets, the basic floral grain-bearing structures, are arranged in an alternate-distichous pattern along the central axis (the rachis). Inflorescence architecture, including both the total number of initiated spikelets and the proportion that ultimately survive to set grain, strongly influences grain yield potential (Arisnabarreta and Miralles, 2008; Kamal *et al*., 2022a, b; Shanmugaraj *et al*., 2023). Barley, possessing an indeterminate inflorescence structure, initiates a large number of spikelet primordia during the early reproductive development, while the end of the spikelet primordia initiation along the rachis marks the maximum yield potential (MYP) stage (Koppolu and Schnurbusch, 2019; Thirulogachandar and Schnurbusch, 2021). However, a substantial fraction of these primordia degenerate before anthesis, particularly in the apical region, resulting in the pre-anthesis tip degeneration (PTD) (Huang *et al*., 2023; Shanmugaraj *et al*., 2023). Both MYP and PTD, as well as final grain number, differ among barley genotypes (Kamal *et al*., 2022a, b; Thirulogachandar and Schnurbusch, 2021). Therefore, understanding the physiological and developmental factors influencing spike PTD may ultimately help improve grain yield.

Recent research, using genetic and transcriptomic approaches, has highlighted the critical role of early chlorophyll accumulation in developing barley inflorescences (Huang *et al*., 2023; Shanmugaraj *et al*., 2023). For example, barley CMF4 (HvCMF4), a CCT-motif family protein, was identified as a key regulator orchestrating chloroplast development, light signaling, and vascular differentiation in the developing inflorescence. Knockout of *HvCMF4* reduced the overall spike chlorophyll accumulation, particularly in the apical region of the developing inflorescence, thereby impairing spike development. This phenotype was further recapitulated by applying photosynthesis inhibitors (norflurazon and lincomycin) to the plant, underscoring the important role of PAIG in supporting proper inflorescence development (Huang *et al*., 2023). Complementary transcriptomic analyses in barley showed that developing spikes, even though covered by several layers of leaf sheaths and exposed to dark conditions, express numerous chloroplast biogenesis, chlorophyll biosynthesis, and photosynthesis-related genes long before emergence (Shanmugaraj *et al*., 2023). Together, these findings highlight PAIG as an overlooked yet potentially important developmental cue, suggesting that the timing and spatial patterning of greening within developing spikes may be functionally linked to spikelet survival, reproductive development, and overall reproductive success.

Although light is known to influence adult inflorescence and grain development after heading, it is unclear whether immature, developing spikes can perceive light, particularly for initiating PAIG, while still being enclosed within leaf sheaths. The extent to which PAIG depends on light and whether limiting light affects spikelet survival or reproductive development remain unknown. These knowledge gaps highlight the need for a mechanistic understanding of PAIG and its developmental function. Despite recent discoveries that barley spikes initiate chlorophyll biosynthesis well before head emergence (Huang *et al*., 2023; Shanmugaraj *et al*., 2023), the timing, spatial progression, and physiological relevance of this process have not been systematically examined. Although photosynthesis inhibitors have been applied to whole plants in attempts to inhibit PAIG (Huang *et al*., 2023), no studies have experimentally disrupted PAIG without affecting overall plant growth to directly test its role in spikelet initiation, survival, or fertility, nor has natural variation in PAIG been linked to genotypic differences in spikelet survival. To address these gaps, we combined high-resolution imaging, chlorophyll quantification, a non-destructive dark-treatment approach, and a diverse barley panel to characterize PAIG and determine its developmental significance. Our findings reveal that early greening is a critical physiological cue supporting meristem viability and reproductive success, thereby establishing PAIG as a previously unrecognized target for improving spike fertility and yield potential in barley.

## Materials and methods

### Plant material and growth conditions

#### Greenhouse experiments

Greenhouse experiments (photoperiod: 16 h/8 h, light/dark; temperature: 20 °/16 °C, light/dark; light intensity: 250 μmol m^−2^ s^−1^; relative humidity: ∼70%) were conducted at the Leibniz Institute of Plant Genetics and Crop Plant Research (IPK) between 2023 and 2025. For both phenotyping and dissection of immature spikes experiments, two-rowed barley cv. Bowman (hereafter Bowman) grains were germinated in a 96-well planting tray for 2 weeks, vernalized at 4 °C for 2 weeks, acclimatized at 15 °C for a week, and finally potted into 9 cm pots until maturity or until sample was collected. Hakaphos red 8+12+24 (Compo Expert) fertilizer was applied 14 d after potting, and Hakaphos blue 15+10+15 (Compo Expert) fertilizer was applied in booting stage.

#### Field experiments

Both phenotyping and dissection experiments were done in field condition at IPK in 2025. The field containing natural loamy soil was manually watered and fertilized similar to the greenhouse. Twenty genotypes of six-rowed spring barley were selected from the ∼350-barley diversity panel of Federal Ex-situ Gene Bank hosted at IPK. The selection was based on the spikelet survival percentage data available from Huang *et al*. (2023) and Kamal *et al*. (2022b) (Supplementary Table S1). Barley grains were germinated in jiffy pots in greenhouse conditions (March 2025). Two-week-old seedlings along with the jiffy pots were transferred to the field conditions with uniform distance of 10–12 cm between each plant, and a distance of 20–25 cm was maintained between each row, with each row containing 10 plants. Spike samples were collected by dissection upon reaching the right stage, and for phenotyping plants were grown until maturity.

### Phenotyping

All phenotypic data from greenhouse-grown plants were collected from the main culm. Final spikelet number was recorded between heading and the grain-filling stage, while grain number, dry biomass, and tiller number were measured at harvest. For plants grown under semi-field conditions, the first three spikes were averaged to determine spikelet number between heading and grain filling, and the same spikes were subsequently used to assess grain number after harvest. The MYP, defined as the total number of spikelet primordia initiated per inflorescence, was assessed by counting spikelet primordia in immature inflorescences collected from both dark-treated and control plants at the Waddington (W) 4.5–5 developmental stage (Waddington *et al*., 1983). Spikelet survival and spike fertility were calculated as follows:


$$\mathrm{Spikelet}\;\mathrm{survival}\;(\text{\%})=\;\left(\frac{\mathrm{Final}\;\mathrm{spikelet}\;\mathrm{number}}{\mathrm{Potential}\;\mathrm{spikelet}\;\mathrm{number}\;\mathrm{at}\;\mathrm{MYP}}\right)\times 100$$

$$\mathrm{Spike}\;\mathrm{fertility}\;(\text{\%})=\left(\frac{\mathrm{Grain}\;\mathrm{number}}{\mathrm{Total}\;\mathrm{spikelet}\;\mathrm{number}}\right)\times 100$$

### Treatment and experimental design

For the dark treatment (DT) experiments, three treatment groups (DT_W2–10_, DT_W2–5.5_, and DT_W4.5–10_) were included along with a control. To prevent light from reaching the developing inflorescence, we developed a method using an aluminum foil strip to directionally shield the targeted region without interfering with overall plant growth or photosynthesis (Supplementary Figs S1). Prior to applying the treatment, plants at comparable developmental stages were dissected to determine the precise position of the internode bearing the developing inflorescence (Supplementary Fig. S1A). Based on this position, an aluminum foil strip (1–1.5 cm wide) was gently wrapped around the leaf sheaths at the first internode on the main culm (Supplementary Fig. S1B). The strip was wrapped in a circular manner to ensure complete coverage of the developing inflorescence and was positioned to cover approximately 2–3 cm above and below the spike position depending upon the stage and size of the inflorescence (Supplementary Fig. S1C). Further, untreated reference plants of the same developmental stage were monitored to track the exact position of the inflorescence, and the foil wrap was adjusted as needed to maintain full coverage throughout development. Additionally, one to two treated plants were periodically dissected to confirm that the developing inflorescence remained fully shielded. The aluminum wrap was applied loosely to avoid any physical restriction to the developing inflorescence. This method allowed passive gas exchange and did not damage surrounding tissues, thereby preventing any direct or indirect effects on overall plant development.

#### Temperature measurements

To measure the local temperature beneath the aluminum-foil cover and around the developing inflorescence, the position of the immature spike at ∼W4.5 was first identified as mentioned earlier (Supplementary Fig. S2A). A digital thermometer with a long probe was then positioned so that the probe tip rested exactly where the spike was located (Supplementary Fig. S2B), after which the aluminum foil was wrapped around the leaf sheaths together with the thermometer probe (Supplementary Fig. S2C, D). This allowed measurement of the ambient temperature beneath the foil cover (Supplementary Fig. S2). Temperature readings were recorded every 2 h throughout the day while the plant remained at the W4.5 stage. Three biological replicates with aluminum-foil covers were monitored, and an additional three uncovered control replicates were used to measure ambient temperature conditions.

### Chlorophyll measurements

For chlorophyll measurements the immature spikes at W4.5–5 and W6 stages were collected by manually dissecting the main culm and further the tissue was frozen immediately using liquid nitrogen in a 2 ml Eppendorf tube. For each biological replication, ∼15 spikes (W4.5–5) and ∼10 spikes (W6) were pooled. Sample collection was done between 11.00 h and 14.00 h. Chlorophyll concentration was measured according to Porra *et al*. (1989). After grinding the tissue samples into fine powder, 1 ml of methanol was added followed by centrifugation at 14 000 *g* for 2 min. Supernatant was collected in clean Eppendorf tubes labelled as set 1. In the remaining supernatant, 1 ml of methanol was added again, followed by centrifugation and supernatant collection in the second set of Eppendorf tubes. The final sample was prepared by taking 600 μl of supernatant from each set, making the final volume 1200 μl. From the final volume, 800 μl was used for the downstream spectrophotometry measurements. The measurements were taken at two wavelengths, i.e. 652 nm and 665.2 nm. Final chlorophyll *a*+*b*, chlorophyll *a*, and chlorophyll *b* concentrations were calculated as follows:


$$\mathrm{Chlorophyll}\;a\;=\;18.22\;A_{665.2}-\;9.55\;A_{652.0}$$

$$\mathrm{Chlorophyll}\;b\;=\;33.78\;A_{652.0}-\;14.96\;A_{665.2}$$

$$\mathrm{Chlorophyll}\;(a+b)\;=\;24.23\;A_{652.0}+\;3.26\;A_{665.2}$$

### Microscopy

Chlorophyll autofluorescence in immature spikes was examined using confocal laser scanning microscopy. Immature inflorescences at developmental stages W4.5–W8.5 were dissected and embedded in 7% agarose within a flat-bottom mold. After solidification, agarose blocks were removed and sectioned into 80–90 µm slices using a vibrating blade microtome (Leica VT1000 S). For earlier stages (W2–W3.5), immature spikes were mounted directly on microscope slides in water and coverslipped. Autofluorescence was captured on an LSM780 confocal laser scanning microscopy system (Carl Zeiss MicroImaging) using a 640 nm laser line and emission from 650 nm to 720 nm.

### Pollen viability

Pollen viability was evaluated using the Amphasys® impedance flow cytometer method. Spikes from all DT and control group plants grown in the greenhouse were collected when anthers were near anthesis. Outer floral organs (lemma and palea) were carefully removed to expose mature, yellow, swollen anthers close to dehiscence. For each replicate, three anthers from a single spikelet were collected prior to opening and transferred into Eppendorf tubes containing Amphasys® buffer 11. Samples were filtered through 100 µm filter heads and analysed by impedance flow cytometry. Pollen was passed through a 120 µm chip, where an electric field was applied, and impedance phase shifts were recorded. Viable and dead pollen were distinguished using a gating threshold calibrated with dead reference samples prepared by boiling pollen at 100 °C for 1 h. The resulting pollen viability data coming from four to eight biological replicates were subsequently used for downstream analyses.

### Tissue collection, RNA isolation, and RT-qPCR

For RT-qPCR analysis, two-rowed barley cv. Bowman was used. Immature spike tissues at developmental stages W6 and W8 were collected from control and dark-treated plants, with approximately ten spikes (W6) or three spikes (W8) pooled per biological replicate. For pathogen-related gene expression analysis, a segment of the second leaf from 2-week-old barley plants was placed on 1% water agar plates with the adaxial side facing upward and evenly inoculated by blowing barley yellow rust spores (*Puccinia striiformis* f. sp. *hordei* race 24) through a settling tower. Inoculated leaves were incubated at 12 °C (8 h night)/16 °C (16 h day) for 2 weeks post-infection and served as a positive control for pathogen-responsive gene expression. All samples were harvested within the same time window (11.00 h to 14.00 h) to minimize diurnal effects. Total RNA was extracted using TRIzol (Thermo Fisher Scientific) and precipitated with 2-propanol, followed by DNase I treatment (New England Biolabs, M0303L) to remove genomic DNA contamination. First-strand cDNA was synthesized from total RNA using the SuperScript III Reverse Transcriptase Kit. Quantitative real-time PCR was performed with SYBR Green Master Mix on an ABI Prism 7900HT (Thermo Fisher Scientific). Three technical replicates were conducted for each of three biological replicates, and two barley house-keeping genes, *HvGAPDH*, encoding glyceraldehyde-3-phosphate dehydrogenase, and *HvActin*, were used as the internal reference genes. Primer sequences were obtained from previously published studies (Zafari *et al*., 2020; Ghouili *et al*., 2022; Huang *et al*., 2023) and are listed in Supplementary Table S2.

### Statistical analysis

All the data analysis and figure generation in this study was done using RStudio Software. Packages such as ggplot2, dplyr, and ggpubr were used for all the data processing and analysis. An unpaired Student’s *t*-test was performed for all comparisons to check the significance of the data. Pearson’s correlation was performed to assess linear relationships between traits.

## Results

### Initiation of greening in the immature inflorescences

**Fig. 1.**
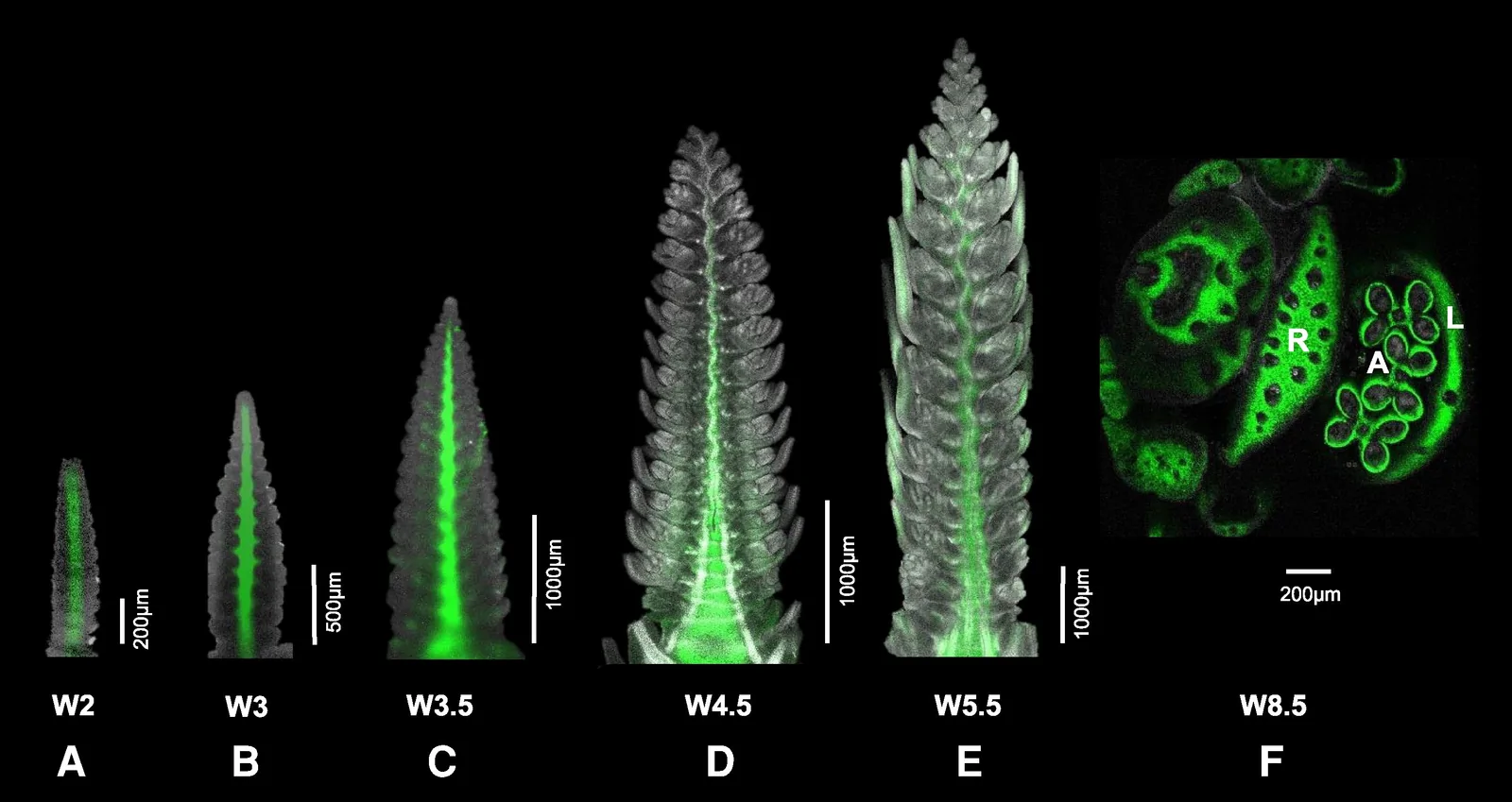
Distribution of chlorophyll in the developing inflorescence, visualized by auto chlorophyll fluorescence at different Waddington developmental stages: W2 (A), W3 (B), W3.5 (C), W4.5 (D), W5.5 (E), and W8.5 (F). A, anther; L, lemma; R, rachis.

**Fig. 2.**
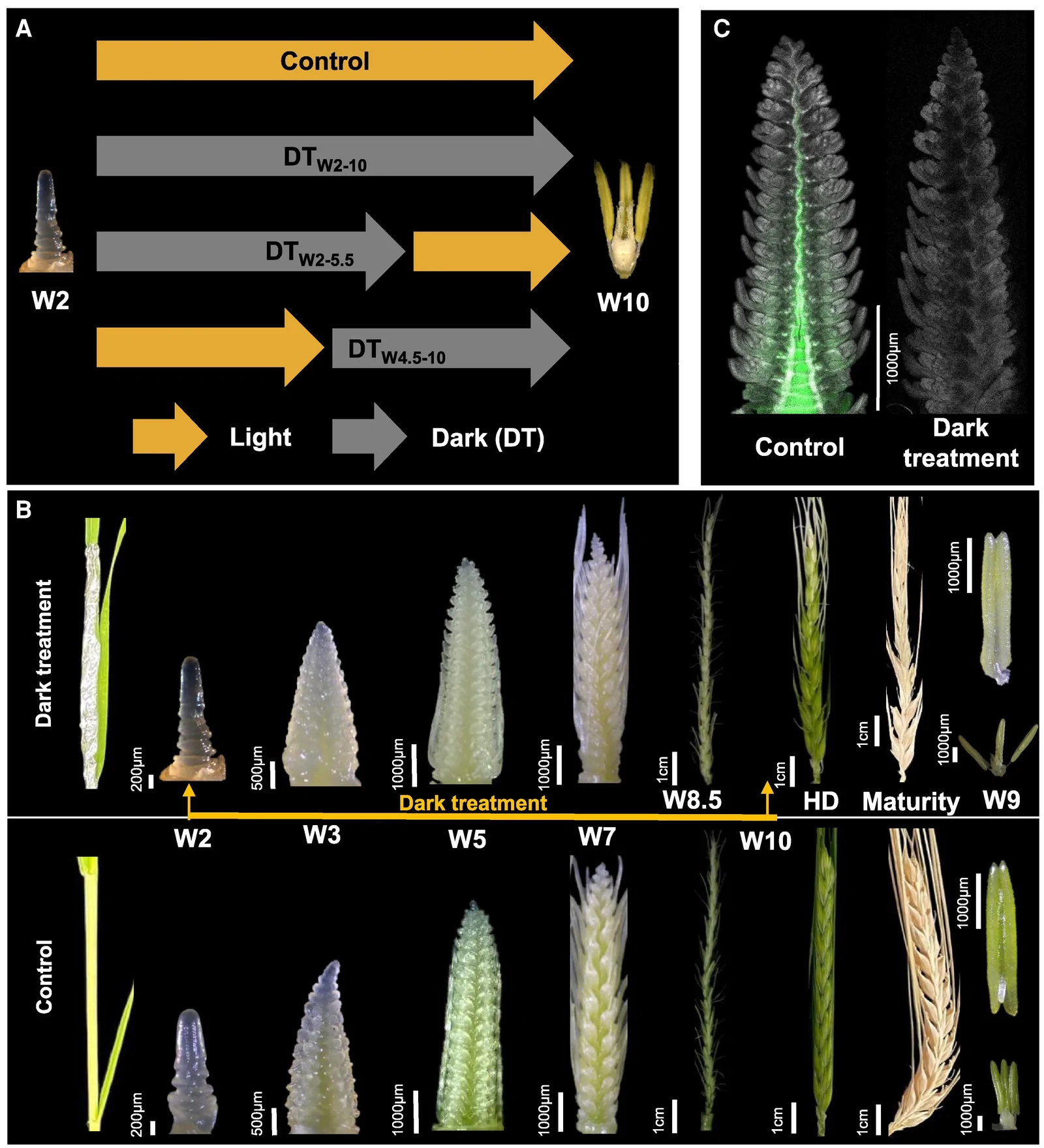
Effects of dark treatment on developing inflorescences. (A) Schematic representation of the treatment groups and their respective treatment phase. (B) Images of the developing inflorescences at different developmental stage between W2 until maturity and anther at W9 from dark treated and control treatments. (C) Chlorophyll autofluorescence in control (left) and dark-treated (right) inflorescence meristems at W4.5.

**Fig. 3.**
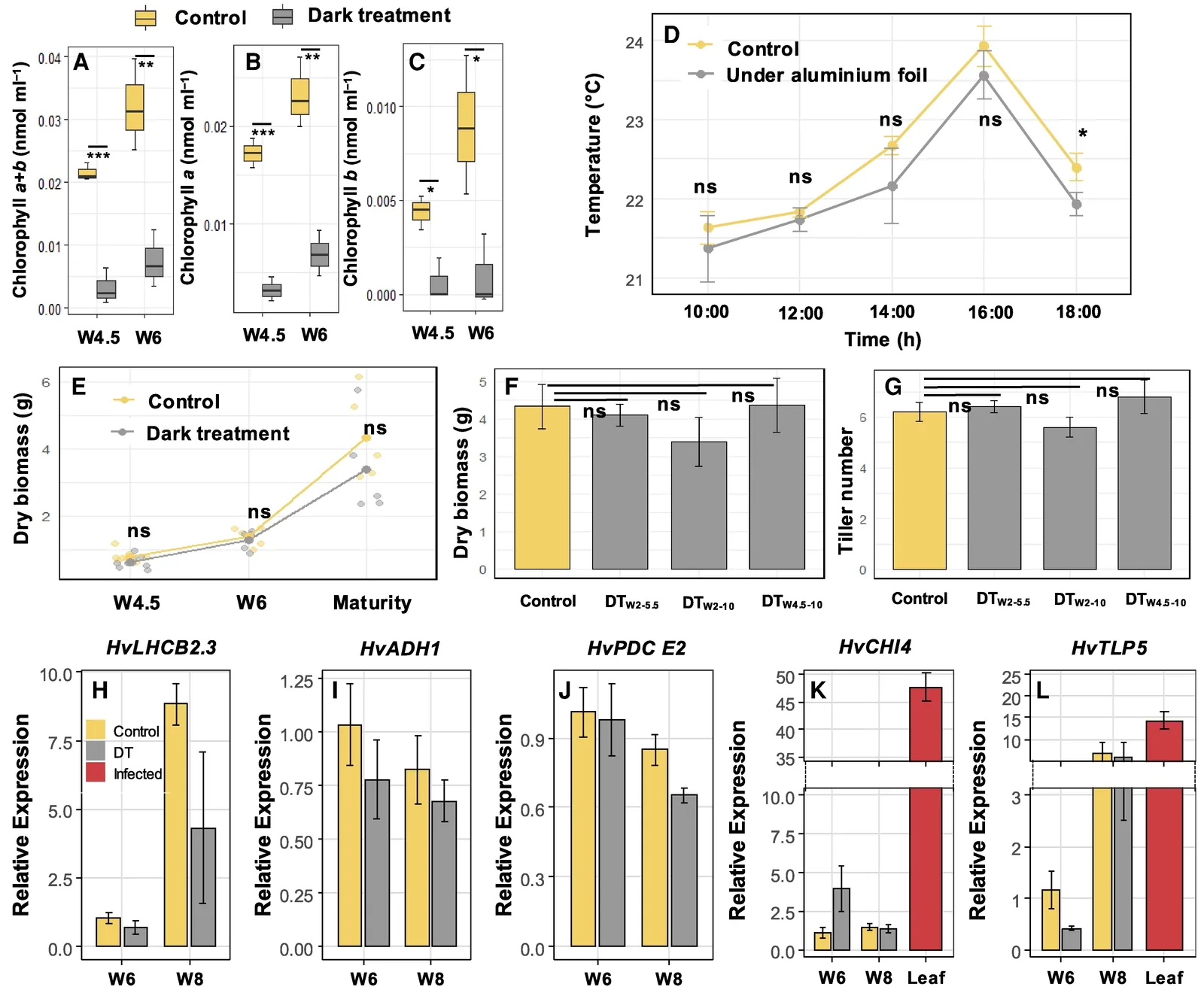
Physiological and molecular responses to dark treatment. (A–C) Chlorophyll concentration in immature spike tissues: total chlorophyll (*a*+*b*) (A), chlorophyll *a* (B), and chlorophyll *b* (C) (*n*=3 biological replicates; each replicate was from the pool of 10–15 individuals). (D) Temperature measurements beneath aluminum foil covering the developing inflorescence compared with ambient temperature over the course of the day (*n*≥3). (E–G) Comparison between control and dark-treated plants (*n*≥5) for above-ground dry biomass at W4.5, W6, and maturity (E); above-ground dry biomass of the whole plant at maturity (F); and number of tillers per plant at maturity (G). DT_W2–5.5_, dark treatment from stage W2 to W5.5; DT_W2–10_ group, dark treatment from stage W2 to W10; DT_W4.5–10_, dark treatment from stage W4.5 to W10. (H–L) Relative expression of marker genes in inflorescence tissue of control and dark-treated plants at W6 and W8. Bars represent mean ±SE (*n*=3 biological replicates; each replicate was from the pool of 3–10 individuals): (H) *HvLHC2.3*, (I) *HvADH1*, (J) *HvPDC E2*, (K) *HvCHI4*, and (L) *HvTLP5*. Statistical significance was assessed by unpaired Student’s *t*-test. Asterisks indicate *P*-values: *P≤0.05, **P≤0.01, ***P≤0.001; ns, not significant.

To determine the onset and spatial progression of chlorophyll accumulation during the development of immature barley inflorescences, which are surrounded by several layers of leaf sheaths, we examined developing spikes at defined stages according to the Waddington scale (Waddington *et al*., 1983). Observations for PAIG started at Waddington stage 2 (W2), a critical point around which the shoot apical meristem transits from the vegetative to the reproductive phase. At W2, a clear chlorophyll autofluorescence signal was already detectable within the immature spikes, indicating the initiation of chlorophyll biosynthesis in the developing rachis area (Fig. 1A). However, greening was not visible to the naked eye at this stage (Fig. 2B). The intensity of the visible autofluorescence signal increased over time (Fig. 1A–F), showing even stronger signal by stage W4.5 (Figure 1D). Spatially, the earliest appearance of chlorophyll was localized mainly to the central portion of the developing meristem, corresponding to the developing rachis region (Fig. 1A–E). As development proceeded, chlorophyll accumulation expanded to the spikelet primordia and subsequently the emerging floral organs, such as the awns, lemma, and palea (Fig. 1D–F). Within the rachis, a distinct acropetal gradient of chlorophyll distribution was persistently observed; the basal region of the immature spike exhibited the strongest autofluorescence signal, which gradually reduced toward the distal tip, eventually disappearing altogether (Fig. 1D, E). Further quantitative analysis of total chlorophyll content in whole spikes supported these imaging results. Increase in chlorophyll levels was detected between stages W4.5 and W6 (Fig. 3A–C), corroborating the visual evidence of enhanced greening and suggesting that chlorophyll biosynthesis accelerates during this developmental window. These results demonstrate that chlorophyll accumulation begins at the earliest stage of inflorescence development and intensifies as inflorescence development progresses.

### A non-destructive method to prevent pre-anthesis inflorescence greening using the dark treatment

We developed a technique to inhibit PAIG by covering the leaf sheaths surrounding the immature inflorescence with aluminum foil strips (Fig. 2B; Supplementary Fig. S1), as described in detail in ‘Materials and methods’. This dark treatment (DT) was applied to three distinct experimental groups (Fig. 2A). In the DT_W2–10_ group, the dark treatment began at developmental stage W2 and maintained until W10, marking the completion of inflorescence development until anthesis. In the DT_W2–5.5_ group, the treatment was applied from W2 to W5.5, approximately the midpoint of inflorescence development in barley; after W5.5, the covers were removed and the plants were exposed to normal greenhouse light conditions. In the DT_W4.5–10_ group, the treatment was applied from W4.5 to W10. A control group was continuously exposed to light throughout development. Meristems were dissected from the DT_W2–10_ and control groups across various developmental stages (W2–W10). From as early as stage W3–3.5, immature spikes from DT plants appeared pale and lacked visible chlorophyll compared with those of the control plants. This pale phenotype persisted throughout later stages of inflorescence development (W4+). Similarly, developing anthers from DT inflorescences exhibited no visible chlorophyll accumulation (Fig. 2B). These pale DT spikes also showed hardly any chlorophyll autofluorescence, indicating a clear decline of chlorophyll (Fig. 2C). Quantification of chlorophyll pigments at stages W4.5 and W6, which correspond to the mid-phase of inflorescence development, a stage of reaching maximum yield potential and initiation of pre-anthesis tip degeneration, confirmed a significant reduction in chlorophyll *a*+*b*, chlorophyll *a*, and chlorophyll *b* levels in DT spikes at both stages (Fig. 3A–C). To further assess the molecular impact of light deprivation, we measured the transcript levels of the gene *HvLHCB2.3*, a light-harvesting chlorophyll *a*/*b*-binding protein and an established marker for light response (Teramoto *et al*., 2002). The expression of *HvLHCB2.3* was sharply reduced in DT spikes compared with control spikes (Fig. 3H), providing additional evidence that DT effectively blocked light perception, chlorophyll biosynthesis, and the expression of photosynthesis-related genes.

To test whether the aluminum cover also affects the local temperature around and below the cover, the temperature beneath the foil was measured using a thermoprobe placed below the cover at multiple time points during the day (Fig. 3D; Supplementary Fig. S2). No significant temperature differences were observed between dark-treated and control plants, indicating that the treatment did not alter the local temperature around the developing inflorescence (Fig. 3D). In addition, agronomic traits, such as total tiller number at maturity, and dry biomass at different developmental stages and at maturity, were recorded for all treatment groups. The dry biomass did not change between DT and control plants across different developmental stages (Fig. 3E). In addition, for different treatment groups at maturity the dry biomass remained unchanged when compared with control (Fig. 3F). Furthermore, no significant differences were detected in the tiller number between the dark-treated and control plants (Fig. 3G), confirming that the DT specifically inhibited spike greening without affecting overall plant growth or development. Because wrapping developing inflorescences with aluminum foil could potentially impose unintended stress—such as hypoxia or increased susceptibility to pathogen infection due to elevated humidity, despite gas exchange not being completely restricted—we examined the expression of selected stress marker genes in spike tissues collected from control and dark-treated plants at W6 and W8. Hypoxia-related marker genes included *Alcohol dehydrogenase 1* (*HvADH1*) and *Pyruvate dehydrogenase complex E2* (*HvPDC E2*) (Peng *et al*., 2001; Kürsteiner *et al*., 2003), while defense-related pathogenesis-associated markers included *Chitinase IV precursor* (*HvCHI4*) and *Thaumatin-like protein* (*HvTLP5*) (Lambertucci *et al*., 2019; Sabir *et al*., 2025) (Fig. 3I–L). No significant differences in the expression of *HvADH1* or *HvPDC E2* were detected between control and dark-treated spikes at either the W6 or W8 stage (Fig. 3I–J), indicating that the treatment did not induce any hypoxic stress in the developing inflorescence. Similarly, *HvCHI4* and *HvTLP5* expression levels did not differ significantly between control and dark-treated spikes at either developmental stage (Fig. 3K, L). In contrast, barley yellow rust (*Puccinia striiformis* f. sp. *hordei* race 24) used as positive controls, showed strong expression of both *HvCHI4* and *HvTLP5* compared with spike samples (Fig. 3K, L). Importantly, our marker gene results demonstrate that the aluminum-cover dark treatment did not trigger hypoxia or pathogen-associated defense responses, showcasing that the observed inhibition of spike greening was not attributable to unintended physiological stress effects. Taken together, this study establishes a simple, non-destructive, and effective method to inhibit greening in immature inflorescences by preventing light exposure using a simple aluminum-cover technique.

### Inhibition of pre-anthesis inflorescence greening impedes floral survival

**Fig. 4.**
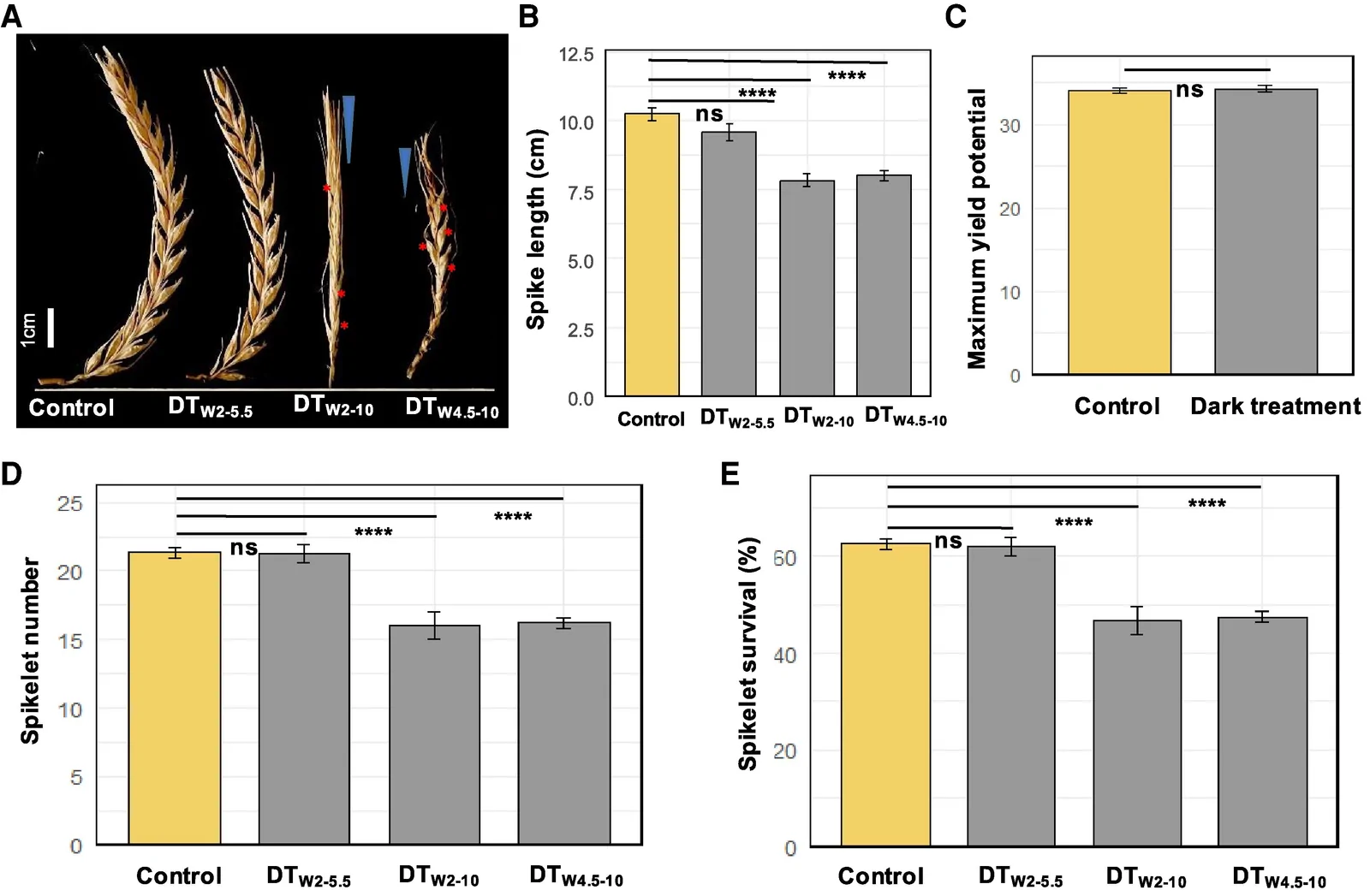
Effects of dark treatment on spike development. (A) Images of mature spikes from different treatment groups. Asterisks indicate the grain-bearing spikelets and blue arrowheads indicates the region of pre-anthesis tip degeneration. (B) Spike length of spikes from different treatment groups taken at maturity (*n*≥5). (C) Maximum yield potential (MYP) of control and dark-treated spikes at W4.5 (*n*=10). (D, E) Comparison between treatment groups at maturity for spikelet number per spike (D) and spikelet survival percentage per spike (E) (*n*≥9). Statistical significance was assessed by unpaired Student’s *t*-test. Asterisks indicate *P*-values: ****≤0.0001; ns, not significant. DT_W2–5.5_, dark treatment from stage W2 to W5.5; DT_W2–10_ group, dark treatment from stage W2 to W10; DT_W4.5–10_, dark treatment from stage W4.5 to W10.

To further evaluate whether the inhibition of PAIG had any influence on reproductive development, we analysed the effect of the DT on spikelet initiation and survival dynamics. Morphological inspection of mature spikes revealed clear phenotypic differences between treatments. Spikes from DT_W2–10_ and DT_W4.5–10_ plants at maturity appeared noticeably smaller, weaker, and with very few grains compared with control spikes, whereas those from DT_W2–5.5_ plants could retain fertile spikelets very efficiently and therefore appeared similar to controls (Fig. 4A, B). To quantify these visual differences, the final spikelet number and spike length was recorded at maturity. While DT_W2–10_ and DT_W4.5–10_ groups exhibited a significant reduction in both spike length and final spikelet number (∼5–6 spikelets) compared with control plants, the earlier DT_W2–5.5_ treatment showed no significant difference in either trait relative to the control (Fig. 4B–D), indicating that light exposure during the later developmental period, that is W4.5–10, is essential for floral success.

Further, the MYP, defined as the total number of spikelet primordia initiated per inflorescence, was determined for immature inflorescences collected from both dark-treated and control plants at the W4.5–5 developmental stage. This stage marks the period when spikelet initiation reaches its peak in barley, making it suitable for assessing potential developmental alterations. Importantly, MYP did not differ significantly between any dark-treated and control spikes (Fig. 4C), proving that the number and rate of spikelet primordia initiation is independent of the greening process in the developing spike.

Spikelet survival was then calculated as the proportion of spikelets that survived at heading relative to the total number initiated. While DT_W2–10_ and DT_W4.5–10_ treatments showed distinctly increased spikelet mortality compared with controls, the DT_W2–5.5_ treatment exhibited spikelet survival rates almost identical to those of the control plants (Fig. 4E). The reduction in the spikelet number in the plants of DT_W2–10_ and DT_W4.5–10_ groups was therefore confirmed to be due to increased spikelet primordia degeneration. In both groups, grains were primarily formed in the spikelets located in the middle portion of the spike, which represent the most developmentally advanced spikelets (Fig. 4A). This pattern indicates that the dark treatment predominantly affects the survival of apical and basal spikelets, while central spikelets remain relatively less affected. Taken together, these findings reveal that the prolonged absence of light and consequent inhibition of chlorophyll accumulation in developing inflorescences negatively affects floral fate during the later stages of spike maturation. Overall, these results demonstrate that while spikelet initiation is unaffected by the inhibition of greening, sustained dark treatment during later inflorescence development compromises spikelet survival, leading to a reduction in the final spikelet number.

### Natural variation in pre-anthesis inflorescence greening affects spikelet survival

To investigate the effect of variable levels of PAIG among different barley genotypes, a set of 20 diverse six-rowed barley genotypes were selected from a broader diversity panel (Supplementary Table S1). The selection was based on the extent of spikelet survival using already published information (Huang *et al*., 2023), with the top 10 genotypes showing the highest spikelet survival and the bottom 10 genotypes exhibiting the lowest spikelet survival. This selection strategy was guided by previous observations in our study suggesting a possible relationship between PAIG and spikelet survival.

**Fig. 5.**
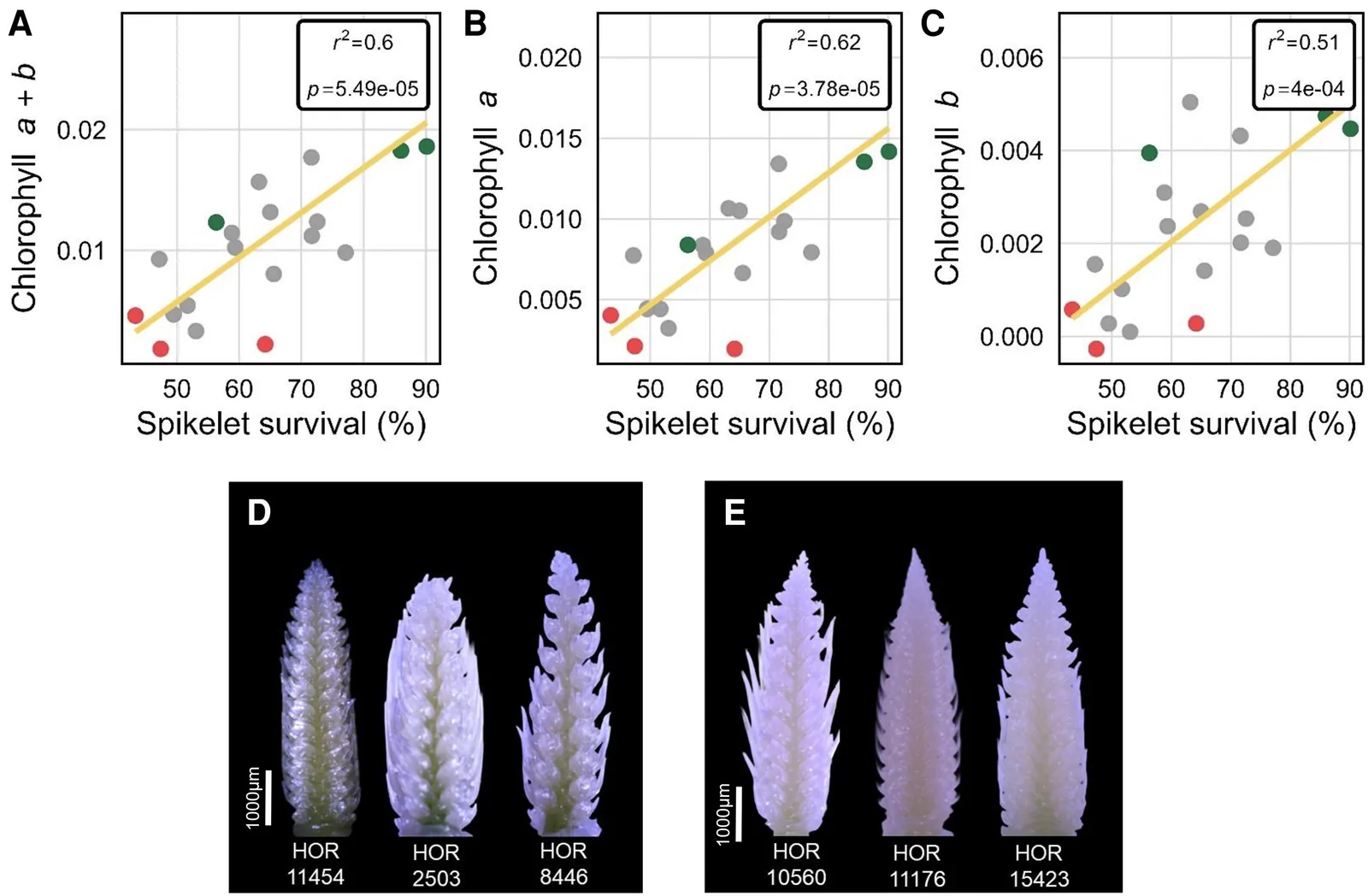
Relationship between chlorophyll content and spikelet survival. (A–C) Pearson’s correlation between spikelet survival percentage and total chlorophyll (*a*+*b*) (A), chlorophyll *a* (B), and chlorophyll *b* (C), quantified at the W4.5–5.5 developmental stage. Green dots indicate the genotypes highlighted in panel (D) (high PAIG group), while red dots indicate the genotypes highlighted in panel (E) (low PAIG group). (D, E) Representative spike images at W4.5 of the high PAIG group (D) and the low PAIG group (E).

Chlorophyll content in immature spikes at the W4.5–5 developmental stage was quantified to assess the degree of greening across genotypes. A significant positive correlation was observed between spikelet survival and chlorophyll content, including total chlorophyll (*a*+*b*), chlorophyll *a*, and chlorophyll *b* (Fig. 5A–C). The chlorophyll *a* and *a*+*b* accounted for ∼60% (*r^2^*=0.62 and 0.6, respectively, *P*<0.001) of the spikelet survival while chlorophyll *b* content accounted for ∼51% (*r^2^*=0.51, *P*<0.001). Genotypes with higher chlorophyll content in their immature inflorescences, particularly within the apical region of the spike, e.g. HOR_11454, HOR_2503, and HOR_8446 (marked in green in Fig. 5), exhibited markedly greater spikelet survival (Fig. 5A–D). In contrast, genotypes with reduced greening in both the whole spike and especially the apical portion, for example HOR_10560, HOR_11176, and HOR_15423 marked in red, displayed a higher incidence of PTD (Fig. 5A–C, E). These results demonstrate that the extent of PAIG, especially in the apical region, is closely linked to the degree of PTD that a spike undergoes, thereby influencing overall spikelet survival.

### Influence of dark treatment on spikelet fertility

As already described, developing anthers normally exhibit visible greening from very early stages of inflorescence development, indicating the onset of chlorophyll accumulation within these tissues. When greening was inhibited by applying the DT to the entire developing spike, including anthers, the anthers appeared pale and completely devoid of visible chlorophyll pigments (Fig. 2B).

**Fig. 6.**
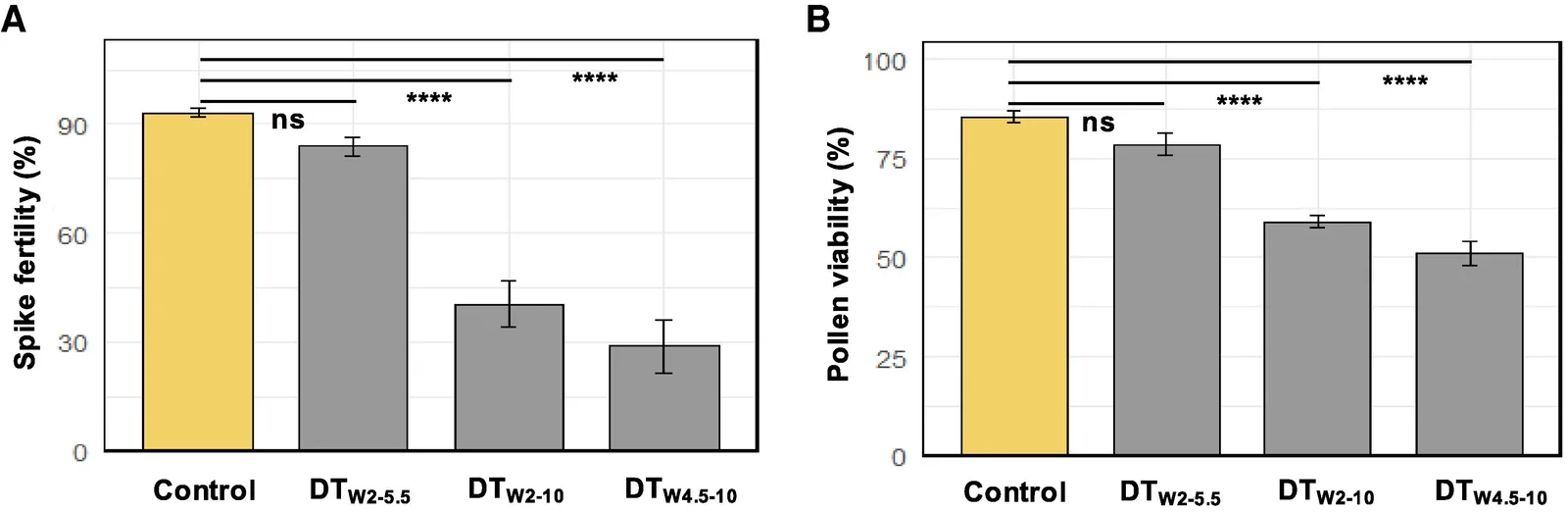
Effects of dark treatment on spike fertility. Comparison between different treatment groups (*n*≥4) for (A) spike fertility percentage and (B) pollen viability percentage. Statistical significance was assessed by unpaired Student’s *t*-test. Asterisks indicate *P*-values: ****≤0.0001; ns, not significant. DT_W2–5.5_, dark treatment from stage W2 to W5.5; DT_W2–10_ group, dark treatment from stage W2 to W10; DT_W4.5–10_, dark treatment from stage W4.5 to W10.

In line with the pale anther phenotype, floral fertility was significantly reduced in the DT_W2–10_ and DT_W4.5–10_ groups compared with the control. In contrast, the DT_W2–5.5_ treatment showed no significant difference in the spike fertility relative to the control spikes (Fig. 6A). This trend closely resembled the observations of spikelet survival reported in the earlier results.

To further investigate the cause of reduced fertility in the DT plants, pollen quality was examined using impedance flow cytometry. As expected, both DT_W2–10_ and DT_W4.5–10_ spikes displayed a significant reduction in pollen viability compared with the control, while DT_W2–5.5_ spikes maintained normal pollen viability (Fig. 6B). These results demonstrate that the suppression of light-dependent greening in developing anthers directly compromises pollen development, leading to impaired fertility. Collectively, these findings indicate a strong association between anther greening and spikelet fertility, suggesting that chlorophyll biosynthesis or light perception within the anther tissues plays a crucial role in maintaining pollen viability and overall reproductive success in barley.

## Discussion

Our study provides new insight into the developmental importance of light-dependent chlorophyll accumulation during early inflorescence development in barley. Here, we demonstrate that (i) PAIG begins at the earliest reproductive stage and follows a defined spatial patterning, (ii) non-destructive inhibition of PAIG through localized DT disrupts spikelet survival and pollen viability, and (iii) natural variation in PAIG across genotypes predicts differences in spikelet survival. Together, these findings highlight a previously underappreciated role of localized photosynthetic competence within developing inflorescence and anthers in shaping reproductive success in cereals.

### Timing and spatial progression of chlorophyll accumulation in immature inflorescences

Chlorophyll biosynthesis begins around W2, which is when the shoot apex shifts to reproductive development (Waddington *et al*., 1983). According to the analyses of chlorophyll autofluorescence, the central developing rachis was the primary site of early greening, which subsequently spread to spikelet primordia and floral organs (Fig. 1). This progression closely parallels cellular differentiation and tissue expansion within the inflorescence (Kirby and Appleyard, 1984), suggesting that chlorophyll biogenesis is developmentally coordinated with the spike’s architectural establishment. Leaves are traditionally considered to be the primary photosynthetic organs in higher plants and major carbon sources (Van Camp, 2005; White *et al*., 2016), whereas reproductive structures are commonly considered carbon sinks that depend largely on leaf-derived photoassimilates (Van Camp, 2005; White *et al*., 2016). However, several pieces of evidence indicate that reproductive organs can make a significant photoassimilate contribution (Aschan and Pfanz, 2003; Raven and Griffiths, 2015; Brazel and Ó’Maoileídigh, 2019), particularly during the later stages of development (Noodén and Penney, 2001). Our findings may extend this concept by demonstrating that immature barley inflorescences accumulate chlorophyll very early during development, even under the limited light that penetrates between the leaf sheaths to reach the developing inflorescence. This early biogenesis raises the possibility that developing spikes possess at least a limited potential for localized photosynthesis or chlorophyll-mediated stress signaling (Trentmann *et al*., 2020; Li and Kim, 2022). However, direct assessment of the photosynthetic machinery and its competence in these tissues will be required in order to better understand the exact role of PAIG in producing photoassimilates and its contribution to spikelet growth and viability.

### Light signal-mediated inflorescence greening

In our study, we demonstrate that the light signal was directly perceived by the developing and sheathed inflorescence. The aluminum foil-mediated DT method developed here provides a simple and non-invasive method to block light perception and further chlorophyll accumulation in the developing spikes without affecting whole plant growth or local temperature. It has been previously reported that altered temperature (Saini and Aspinall, 1982; Prasad and Djanaguiraman, 2014; Jagadish, 2020; Hu *et al*., 2021) or altered development (Willey and Holliday, 1971) can significantly disrupt inflorescence development and increase sterility. In our study, no significant differences were observed in ambient temperature beneath the aluminum foil, aboveground dry biomass, or tiller number (Fig. 3D–G). To exclude unintended physiological stress caused by the foil cover, we analysed hypoxia- and pathogen-responsive marker genes (Fig. 3I–L). *HvADH1* and *HvPDC E2* showed no significant differences between control and dark-treated spikes at W6 or W8, suggesting that hypoxic stress was not induced. Similarly, the defense-related marker genes *HvCHI4* and *HvTLP5* remained unchanged in dark-treated spikes, whereas barley yellow rust-infected leaf samples inoculated with *Puccinia striiformis* f. sp. *hordei* race 24 exhibited strong expression of *HvCHI4* and *HvTLP5*, confirming marker responsiveness. In contrast, expression of the photosynthesis-related marker gene *HvLHC2.3* was strongly reduced in dark-treated spikes compared with controls (Fig. 3H). Collectively, these findings indicate that the observed phenotypes were directly attributable to suppressed chlorophyll biosynthesis in the developing spike and not the result of unintended physiological stress. The pale phenotype of both developing floral structures and anthers from early stages (W3–3.5) onward, together with significant reductions in chlorophyll *a*, *b*, and total chlorophyll levels, underscores the essential role of light perception in promoting plastid differentiation and chlorophyll accumulation. These findings are consistent with the well-established requirement of light-regulated transcriptional and plastid developmental pathways for chlorophyll biosynthesis (Chen *et al*., 2004; Pogson and Albrecht, 2011; Jarvis and López-Juez, 2013; Yuan *et al*., 2017). Consequently, the absence of any light disrupts these coordinated biogenetic processes in developing inflorescence tissues, leading to a persistent chlorophyll-deficient phenotype. In contrast, the initiation of chlorophyll biosynthesis in control spikes indicates that some light can penetrate through the surrounding leaf sheath layers to reach the immature inflorescence, providing a critical cue for chlorophyll accumulation. Although most of the blue and red wavelengths of the visible light spectrum are absorbed by the surrounding leaf sheath tissues, unabsorbed green light may still reach the developing inflorescence. While monochromatic green light is often underappreciated as a signaling cue, several studies have shown that it can trigger specific developmental responses in plants, such as hypocotyl elongation in Arabidopsis, accumulation of geranylgeranylated chlorophylls in barley, and altered developmental rates in wheat (Kasajima *et al*., 2009; Materová *et al*., 2017; Smith *et al*., 2017; Battle *et al*., 2020). Previous work has also demonstrated that light can be transmitted through intercellular spaces or vascular tissues (Sun *et al*., 2003, 2005; Nawkar *et al*., 2023), which may provide sufficient illumination to activate the light-dependent gene networks necessary for early chloroplast development.

### Pre-anthesis inflorescence greening as a physiological determinant of overall reproductive fitness in cereals

Selectively inhibiting the light revealed that prolonged darkening (DT_W2–10_ or DT_W4.5–10_) drastically reduced final spikelet number, whereas early short-term inhibition (DT_W2–5.5_) allowed full retention (Fig. 4D). Interestingly, maximum primordia number remained unchanged (Fig. 4C). Therefore, the reductions in final spikelet number were attributable to increased PTD during later developmental stages (Thirulogachandar and Schnurbusch, 2021; Huang *et al*., 2023; Shanmugaraj *et al*., 2023). PTD is a major determinant of grain number in barley (Thirulogachandar and Schnurbusch, 2021; Kamal *et al*., 2022b; Huang *et al*., 2023) and is strongly influenced by local assimilate supply and developmental stability (Shanmugaraj *et al*., 2023; Huang and Schnurbusch, 2024). However, in both dark-treated groups DT_W2–10_ and DT_W4.5–10_ the surviving spikelets were primarily located in the middle portion of the spike (Fig 4A), suggesting that both PTD and basal abortion may contribute to spikelet loss (Huang and Schnurbusch, 2024; Tamagno *et al*., 2024), a phenomenon that requires further investigation. The pronounced effects of light deprivation suggest that developing spikes may rely on chloroplast-derived cues to support rapid cellular differentiation and establish proper tissue identity. Chloroplasts play a central role in guiding cell fate and tissue differentiation, ensuring that cells develop appropriately for their location and function (Guo *et al*., 2016; Liebers *et al*., 2022; Sierra *et al*., 2023). In cereal spikes, early chloroplast development likely provides signals that coordinate spikelet survival, vascular differentiation, and overall inflorescence architecture, in addition to later supplying photoassimilates once tissues are fully differentiated. This is consistent with evidence that cereal inflorescences can act as both sinks and modest photosynthetic sources at later stages of development (Bort *et al*., 1994; Tambussi *et al*., 2005; Maydup *et al*., 2012; Sanchez-Bragado *et al*., 2014; AuBuchon-Elder *et al*., 2020). Natural variation among 20 barley genotypes further supported this functional link (Fig 5): chlorophyll content in immature spikes explained 50–62% of variation in spikelet survival, and genotypes with low PAIG showed increased tip degeneration, aligning with known genotypic differences in PTD or spikelet survival in barley (Kamal *et al*., 2022a, b). PAIG therefore emerges as a quantitative trait influencing reproductive fitness and offers potential breeding value, especially given the identified quantitative trait loci for spikelet survival and the association of the candidate gene with PAIG (Huang *et al*., 2023). Notably, PAIG is not restricted to barley but is a characteristic feature of several major cereals, including wheat and rye, suggesting that early inflorescence greening represents a conserved developmental strategy that can be exploited as an important trait for crop improvement across *Triticeae* species. Beyond spikelets, we also found that anther greening initiated early during microsporogenesis. As reported earlier, the light signal influences spike fertility (González *et al*., 2003; Ugarte *et al*., 2010; Dreccer *et al*., 2022). Similarly, also in our study, dark-treated developing anthers remained pale (Fig. 2B) and produced largely non-viable pollen, which in turn hampers the overall spike fertility (Fig 6). This indicates that light-dependent chloroplast maturation may support tapetum function, lipid biosynthesis, and microspore metabolism (Ariizumi and Toriyama, 2011; Zhu *et al*., 2020). Similar sterility phenotypes are observed in mutants deficient in endothecium chloroplast development (Zhu *et al*., 2020). Although we propose that the absence of light compromises pollen viability and fertility, this observed effect is unlikely a direct result from of the lack of light on pollen cells, as they do not possess functional photosynthetic organelles. Rather, it is more plausibly that it is an indirect consequence of the DT on the surrounding anther tissues, which contain photosynthetically active plastids and contribute to pollen maturation through metabolic and signaling support. Moreover, potential effects of DT on female reproductive organs and their contribution to spikelet sterility remain to be investigated. Future experiments incorporating recovery assays, or direct morphological analyses of female reproductive organ development will be crucial to fully elucidate the contribution of female defects to spikelet sterility. Also, further studies integrating molecular and biochemical approaches will be essential to establish a mechanistic link between spike/anther greening and reproductive fitness. Collectively, these results establish that light perception and greening in the developing inflorescence of barley and other cereals are not passive traits but integral processes that enhance spikelet retention and pollen fertility, thereby underpinning overall reproductive fitness.

## Supplementary Material

erag216_Supplementary_Data

## Data Availability

The primary data supporting this study were not made publicly available at the time of publication. The data that support the findings of this study are available from the corresponding author upon request.

## Supplementary data

The following supplementary data are available at *JXB* online.

Fig. S1. Dark-treatment procedure used to prevent light exposure to the developing barley inflorescence at approximately W4.5 by wrapping aluminum foil around the leaf sheaths enclosing the spike-bearing internode.

Fig. S2. A step-by-step illustration of the procedure used to measure temperature beneath the aluminum-foil cover placed around the spike-bearing internode.

Table S1. List of 20 spring barley genotypes selected from the IPK diversity panel.

Table S2. Primer sequences used for RT-qPCR analysis in this study.
