# Supplementary material for: Pre-anthesis light signaling of sheathed developing barley inflorescences defines floral fate
Source: J Exp Bot. 2026 May 13;77(17):5807–20. doi: 10.1093/jxb/erag216 (PMC13577744; doi:10.1093/jxb/erag216)
Supplement: Supplementary file 1 [file erag216_supplementary_data.pdf]

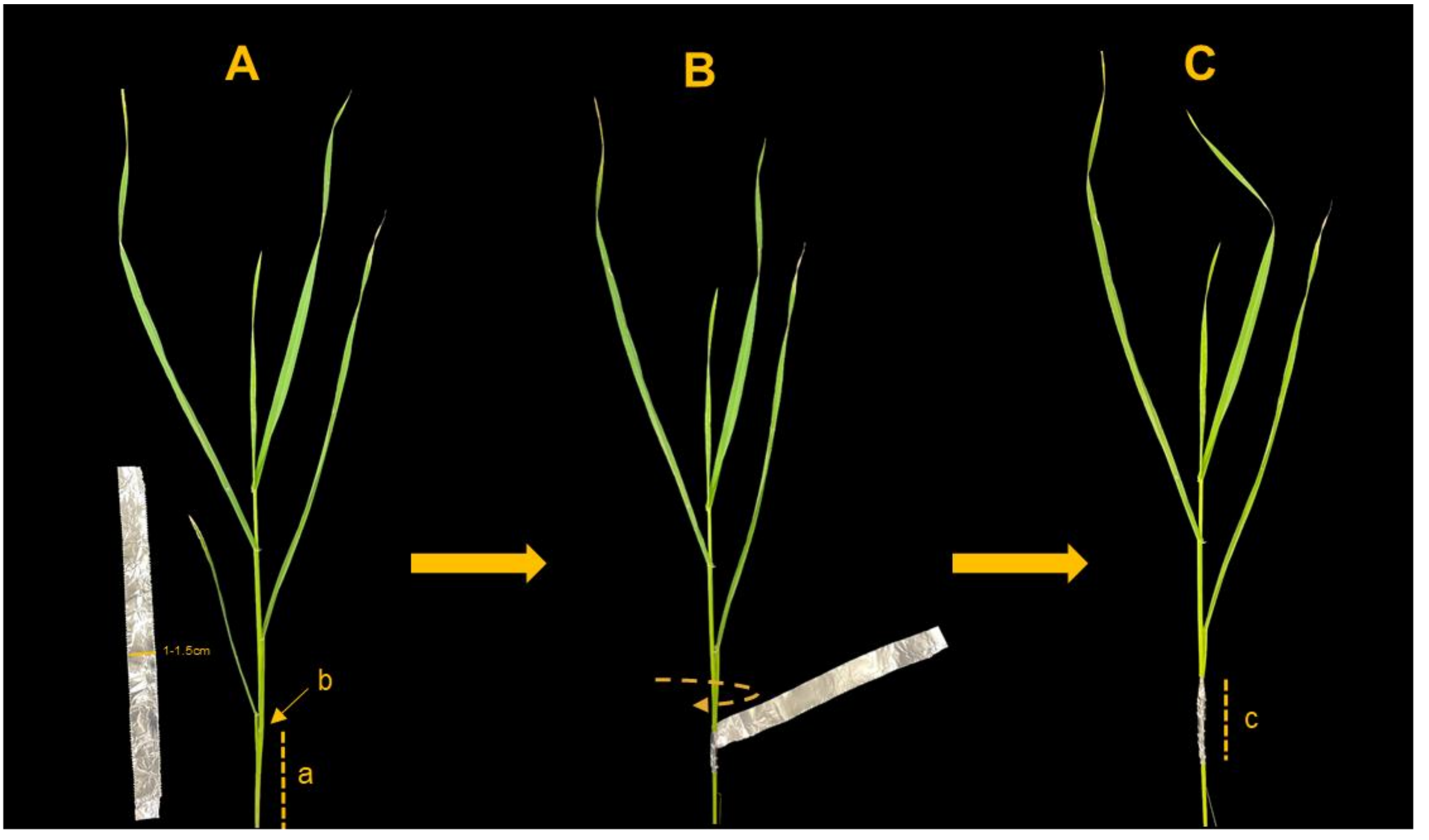

Figure S1 - Dark-treatment procedure used to prevent light exposure to the developing inflorescence at ~W4.5. (A) Identification of the position of the developing spike. (B) Wrapping aluminum foil around the leaf sheaths surrounding the internode at the spike position. (C) Ensuring coverage of the leaf sheaths approximately 2–3 cm above and below the spike to maintain complete darkness. (a) Distance between the internode bearing developing spike and ground, (b) Internode bearing the developing inflorescence, (c) Total area covered by aluminum foil.

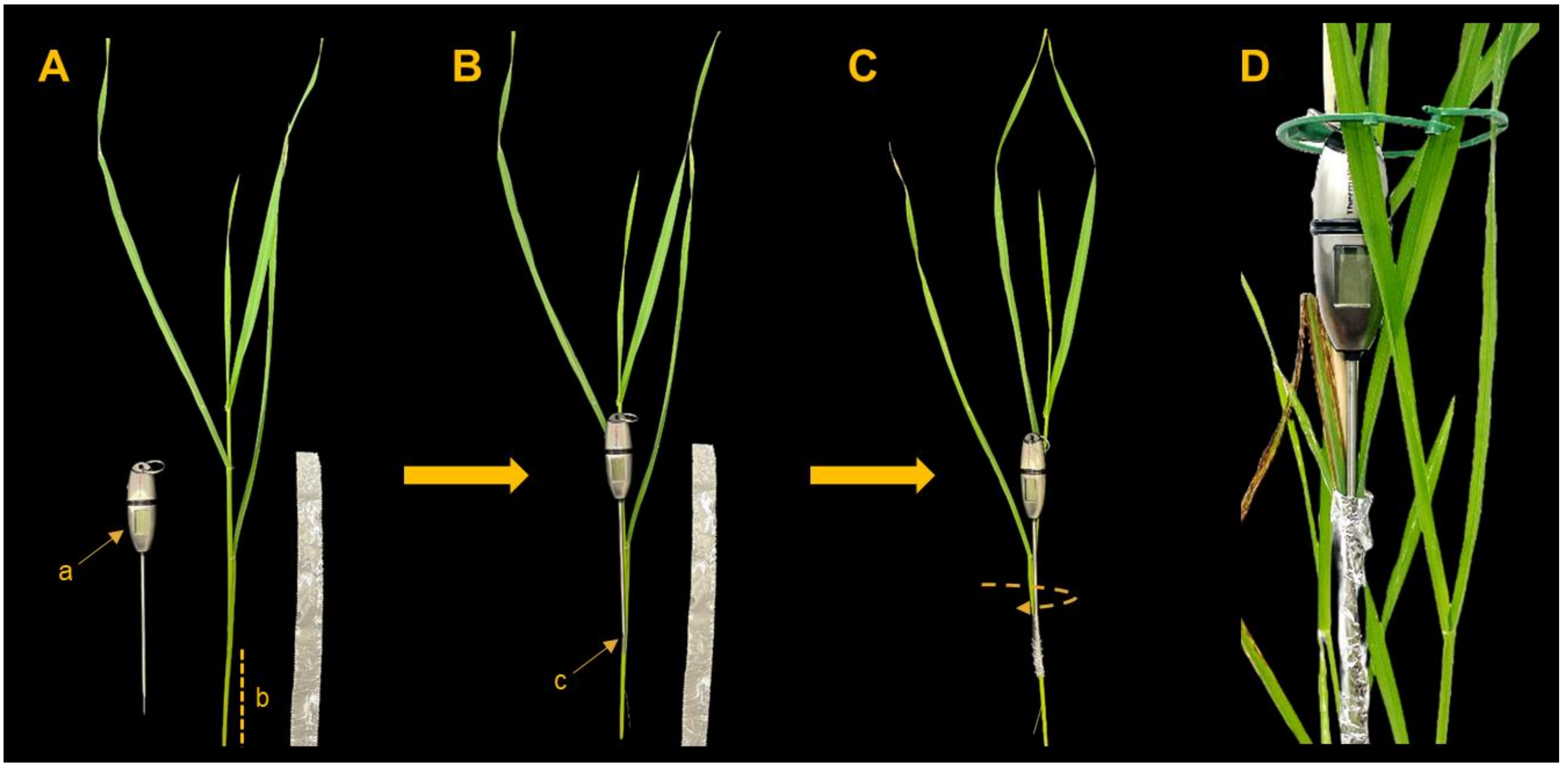

Figure S2: A step by step illustration of the procedure used to measure temperature beneath the aluminum-foil cover. (A) Identification of the position of the developing spike. (B) Placement of the thermometer such that the probe tip sits at the internode where the spike is developing. (C) Wrapping the aluminum foil around the leaf sheath and thermometer probe. (D) Providing a support stick to stabilize the thermometer. (a) Long-probe digital thermometer; (b) distance between the internode bearing the developing spike and the ground; (c) internode bearing the developing spike.

Table S1: List of twenty spring barley genotypes chosen from the IPK diversity panel

Genotype	Country	Genebank_status
HOR_14156	ESP	landrace
HOR_1858	CHN	landrace
HOR_11124	ITA	landrace
HOR_11431	PER	landrace
HOR_4065	USA	cultivar
HOR_11407	CHN	landrace
HOR_15423	PAK	landrace
HOR_11176	TUN	landrace
HOR_10560	ITA	landrace
HOR_14221	NOR	landrace
HOR_8446	IND	landrace
HOR_14433	-	cultivar
HOR_2503	CHN	landrace
HOR_21087	-	landrace
HOR_11454	USA	cultivar
HOR_13870	IRN	landrace
HOR_2403	CAN	cultivar
HOR_2367	CHN	cultivar
HOR_2749	USA	cultivar
HOR_20714	SUN	landrace

Table S2: Primers used in this study for RT-qPCR			
**Gene name**	**Forward (5'-3')**	**Reverse (5'-3')**	**Reference**
HvLHCB2.3	CACACACCATCCTCCAGTTG	CAGGTACTTGGGACGGTCAG	Huang et al., 2023
HvADH1	GATCTGCTCAGGATCAACAC	GTGGAAGTCCCTACGAAATG	Zafari et al., 2020
HvPDC-E2	TGCAGGGATGGAAAGAGCTT	GGTTGGAGCTGCTTCATACG	Zafari et al., 2020
HvCHI4	GGACCAATTCCAGCTACTGC	GACTTGATCCCGTCGAAGAA	Ghouili et al., 2022
HvTLP5	ACTGCCCGCCAACATACTAC	GTGCTGGTCTGGTCATCCTT	Ghouili et al., 2022
HvActin	AAGTACAGTGTCTGGATTGGAGGG	TCGCAACTTAGAAGCACTTCCG	Huang et al., 2023
HvGAPDH	GTGAGGCTGGTGCTGATTACG	TGGTGCAGCTAGCATTTGAGAC	
